# Progressive Comparison of Density Assessment of Alveolar Bone Graft in Patients with Unilateral and Bilateral Cleft

**DOI:** 10.3390/jcm10215143

**Published:** 2021-11-01

**Authors:** Pin-Ru Chen, Yu-Ching Lin, Betty Chien-Jung Pai, Hsiao-Jung Tseng, Lun-Jou Lo, Pang-Yun Chou

**Affiliations:** 1 Craniofacial Research Center, Department of Plastic and Reconstructive Surgery, Chang Gung Memorial Hospital, Chang Gung University, Taoyuan 33302, Taiwan; pinru99@gmail.com (P.-R.C.); lunjoulo@cgmh.org.tw (L.-J.L.); 2 Department of Medical Imaging and Intervention, Chang Gung Memorial Hospital at Keelung, College of Medicine, Chang Gung University, Taoyuan 33302, Taiwan; yuching1221@gmail.com; 3 Craniofacial Research Center, Department of Craniofacial Orthodontics, Chang Gung Memorial Hospital, Chang Gung University, Taoyuan 33302, Taiwan; pai0072@cgmh.org.tw; 4 Clinical Trial Center, Biostatistics Unit, Chang Gung Memorial Hospital, Taoyuan 33302, Taiwan; allebjht@gmail.com

**Keywords:** alveolar bone grafting, alveolar cleft, bone mineral density, density enhancement rate, cone beam computed tomography (CBCT)

## Abstract

(1) Background: Continuing to observe the grafted bone mineral density (BMD) is essential to ensure the success of alveolar bone grafting (ABG) in patients with cleft lip and palate. This study elaborates on three methods that can be used to evaluate the progressive BMD. (2) Methods: Forty patients with unilateral or bilateral clefts receiving ABG were enrolled. Cone beam computed tomography (CBCT) scans were taken at 6 months (T1) and 2 years (T2) postoperatively. In CBCT, measurements were obtained on three different planes using the circle located 1 mm from the adjacent teeth (Method A), the largest circle within the defect (Method B), or the central circle with a diameter of 2 mm (Method C). The BMD was the average density of the three planes and was adjusted by pogonion density. Bland–Altman plots were used to evaluate the agreement of each method. Inter-rater reliability was confirmed by the intraclass correlation coefficient (ICC). (3) Results: For Method A, B, and C, the mean-adjusted BMD (BMD/pogonion density, BMD_a_) was 17.44%, 17.88%, and 17.69%, respectively, at T1 (*p* = 0.495), and 22.51%, 22.87%, and 22.74%, respectively, at T2 (*p* = 0.690); the density enhancement rates were 40.54%, 38.92%, and 43.15% (*p* = 0.382). Significant differences between the BMD_a_ at T1 and T2 were observed (*p* < 0.001, <0.001, and 0.001, for Method A, B, and C, respectively). The volume of the grafted tissue remained stable during T1 and T2, and no significant correlation between density enhancement rate and volume loss was observed. (4) Conclusions: A significant increase in the BMD of grafted tissue was observed in the 2-year postoperative follow-up. The three methods for measuring BMD_a_ via CBCT can be applied in post-ABG evaluations.

## 1. Introduction

Secondary alveolar bone grafting (ABG) is a standard and necessary procedure for treating patients with cleft lip and palate (CLP) [1,2]. ABG serves to stabilize the continuity of the maxillary arch, provide support for tooth eruption, provide bony support for adjacent teeth to ensure long-term periodontal health, support the alar base, and improve speech. If ABG is not performed properly, midface retrusion may be observed when the bone matures [3,4,5].

Traditionally, imaging assessment of ABG outcomes is conducted using two-dimensional radiography and scales, such as the Bergland scale; however, computed tomography (CT)-based studies have indicated that conventional radiographs tend to overestimate bone growth [3,6,7]. Cone beam CT (CBCT) is characterized by a low radiation dose and cost effectiveness; it can be performed using high-quality three-dimensional (3D) image acquisition and reconstruction parameters, including the characteristics of the maxillary alveolar anatomical boundaries; therefore, researchers are increasingly using CBCT for diagnostic and therapeutic evaluations of ABG [8,9,10]. Because the success of a dental implantation is affected by bone quality, assessing bone height and bone mineral density (BMD) is essential before a dental implantation is performed [11]. Many studies have examined bone height; however, few have proposed methods for assessing the BMD of grafted tissue and tracking the progression of BMD.

A new ABG method for Scarpa’s fascia grafts [2] and obtained CBCT images of children with CLP were adopted to prepare for postoperative follow-up. In the present study, we used the aforementioned CBCT images to design a new and easier method for evaluating the BMD of grafted tissue. We proposed three methods for measuring BMD and hypothesized that the three methods would yield similar results. In addition, we compared the density of grafted tissues with adjacent bony tissue in the growth stages and assessed the density enhancement rate in the first 2 years after surgery.

## 2. Patients and Methods

### 2.1. Patient Information and Data Collection

Patients who had nonsyndromic unilateral or bilateral cleft lip and alveolus with or without cleft palate and received ABG at our center between 2016 and 2018 were enrolled. All patients underwent at least 2 CBCT scans after operation. Postoperative image acquisition was performed at two time points, namely 6 months (T1) and 2 years (T2) after ABG. The ABG procedures for all patients were performed by the same senior surgeon as per the protocol of our center, which comprises an iliac cancellous bone graft and the sealing of Scarpa’s fascia to the defect before the bone graft is packed [2,12]. Patients who had syndromic cleft alveolus, who underwent two-stage ABG (performed for bilateral cases), or who exhibited failed grafted tissue were excluded. In total, 40 patients were enrolled in the present study. The demographic and clinical variables for ABG were retrospectively collected through a review of medical charts. All CBCT images were obtained using an i-CAT CBCT scanner (Imaging Sciences International, Hatfield, PA, USA); the parameters for the images are as follows: 120 kVp, voxel size of 0.4 × 0.4 × 0.4 mm^3^, 40 s scan time, and 22 × 16 cm field of view.

### 2.2. BMD Measurement and Volumetric Analysis

**Figure 1:**
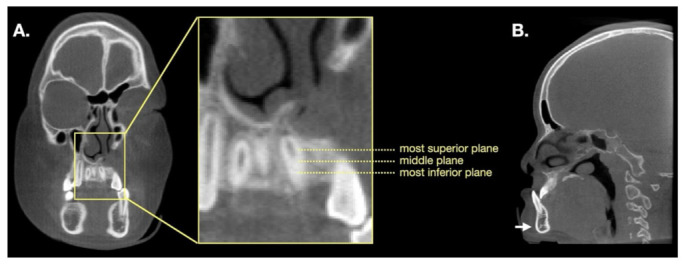
Identification of the selected planes. (**A**) Identified the most superior and inferior planes transecting the grafted tissue on the coronal view image; subsequently, the middle plane of the grafted tissue was identified. (**B**) The pogonion was identified on the sagittal view.

The Picture Archiving and Communication System (PACS) was applied for BMD analysis. Three methods (A, B, and C), which differed by the size of the selected area, were designed to measure Hounsfield units (HUs). For all methods, we first identified the most superior and inferior planes transecting the grafted tissue on the coronal view image; subsequently, the middle plane of the grafted tissue was identified (Figure 1). For the selected plane, the distance between two teeth had to be larger than 2.5 mm in diameter; this was required for all three methods. On each plane, the selected circular area is defined as being located at the midpoint of the line between the centers of the two adjacent teeth. The HU of the selected area could be obtained using PACS.

**Figure 2:**
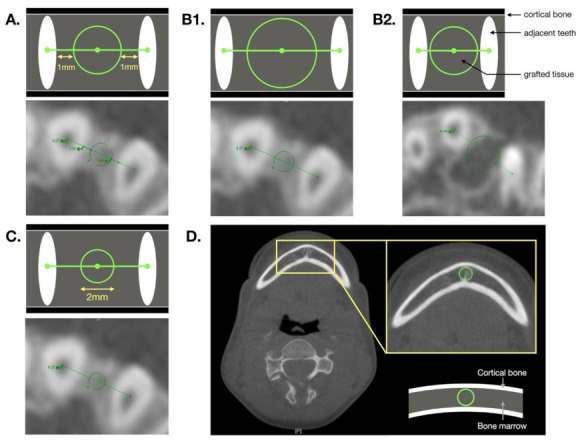
Axial image of grafted area with schemes. (**A**) For Method A, a circle located 1 mm from the adjacent teeth is sketched. For Method B, the largest circular area of grafted tissue is sketched. Two possible conditions apply for Method B. (**B1**) If the distance between two adjacent teeth is greater than the thickness of the alveolar defect, a circle tangent to surrounding cortical bone density is drawn. (**B2**) If the distance between two adjacent teeth is less than the thickness of the alveolar defect, a circle transecting the two adjacent teeth is selected. (**C**) For Method C, a central circle with a diameter of 2 mm is drawn. Note that (**A**), (**B1**), and (**C**) are images of the same patient. (**D**) The largest circular zone within the pogonion bone marrow is used to obtain the pogonion density for calibration.

For Method A, the circular zone located 1 mm from the adjacent teeth was selected. For Method B, the largest circular zone that exactly transected the two adjacent teeth or was tangent to the surrounding cortical bone was selected. For Method C, a circle was drawn with a diameter of 2 mm. Furthermore, the largest circular zone in the pogonion bone marrow was selected to obtain its HU at T1 and T2; this was the reference for calibration (Figure 1 and Figure 2). HU was the reference for BMD and represented the average density of the three planes. We defined the adjusted BMD (BMD_a_, %) using the following formula:

(1) $${\mathrm{BMD}}_{a}\left(\%\right)\;=\;\frac{{\mathrm{HU}}_{\mathrm{selected}\;\mathrm{zone}}}{{\mathrm{HU}}_{\mathrm{pogonion}}}\;\times \;100.$$

The density enhancement rate (%) was calculated using the following formula:

(2) $$\mathrm{Density}\;\mathrm{enhancing}\;\mathrm{rate}\;\left(\%\right)\;=\;\frac{{\mathrm{BMD}}_{\mathrm{aT}2}\;-\;{\mathrm{BMD}}_{\mathrm{aT}1}}{{\mathrm{BMD}}_{\mathrm{aT}1}}\;\times \;100,$$

where BMD_aT2_ is BMD_a_ measured at T2 and BMD_aT1_ is the BMD_a_ at T1.

All the BMD and pogonion density data were measured by one examiner and repeated twice on different dates that were separated by a 3-month interval; the second round of measurements was conducted without reference to the first-round measurements. The average of the six sets of data (two measurements for each of the three planes) was used for the final statistical analysis.

**Figure 3:**
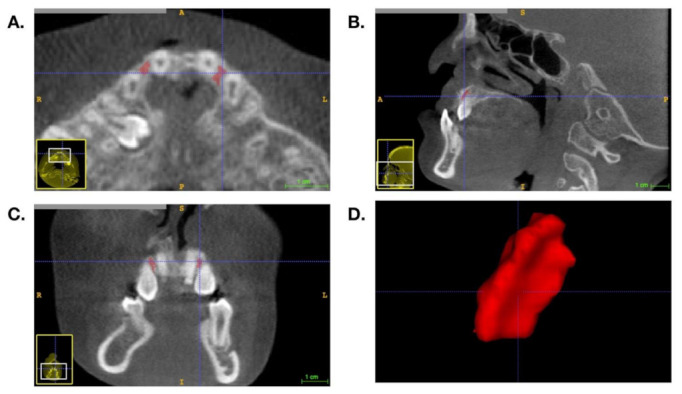
Volumetric analysis of the grafted tissue using ITK-SNAP 3.6.0 open-source software. (**A**) The axial view of the grafted tissue. (**B**) The sagittal view of the grafted tissue. (**C**) The coronal view of the grafted tissue. (**D**) The 3D display of the grafted tissue after segmentation.

As for the volumetric analysis, segmentation of the grafted tissue was performed using ITK-SNAP 3.6.0 open-source software [13]. The grafted tissue was confirmed by axial, coronal, and sagittal views at the same time (Figure 3), and the volume of the segmented area could be displayed.

### 2.3. Statistical Analysis

In the descriptive analysis, continuous variables were summarized by means ± standard deviations, with an independent *t*-test used to compare the means between two groups; a chi-squared test or Fisher’s exact test was used for categorical data. Furthermore, we performed analysis of variance (ANOVA) with repeated measurements to compare the differences between the three methods with respect to BMD_a_ measurements and density enhancement rates; a Friedman test was used for the bilateral group due to a small sample size. To compare the differences in BMD_a_ at T1 and T2, we performed a paired *t*-test or a Wilcoxon signed-rank test for the bilateral group. The correlation between volume loss and density enhancement rate between T1 and T2 was performed using Spearman’s correlation. Bland–Altman plots were used to evaluate the agreement of each method. Intraclass correlation coefficients (ICCs) were calculated as the measure of the intrarater reliability for the three methods. All data were analyzed using SPSS 24.0 (SPSS, Chicago, IL, USA). A *p*-value of less than 0.05 was considered statistically significant by two-tailed tests.

## 3. Results

### 3.1. Subject Characteristics

The study was approved by the relevant institutional review board. In total, 40 patients, who had an average age of 9.45 ± 1.61 years when they received ABG, were enrolled. Among the patients, 60% (*n* = 24) were male and 82.5% (*n* = 33) had unilateral alveolar cleft. The first (T1) and second (T2) follow-up CBCTs were taken at 6.67 ± 0.82 months and 24.04 ± 5.15 months after ABG (Table 1).

### 3.2. Adjusted BMD and Density Enhancement Rate

**Figure 4:**
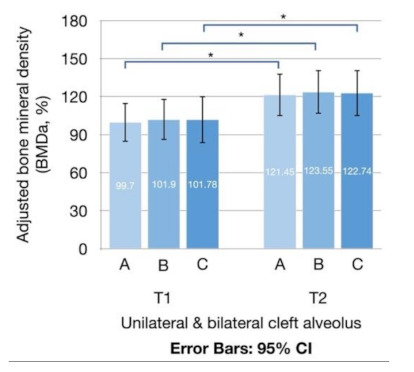
Error bars of adjusted bone mineral density (BMD_a_) of grafted tissue at T1 and T2. Stars indicate significant differences in BMD_a_ between T1 and T2: *, *p* < 0.05.

The mean BMD_a_ of the grafted tissue at T1 were 99.70% ± 49.18%, 101.90% ± 52.05%, and 101.78% ± 59.74% when measured by Method A, B, and C, respectively; at T2, the values were 121.45% ± 53.98%, 123.55% ± 55.52%, and 122.74% ± 58.81% for Method A, B, and C, respectively (Figure 4 and Table 2). When comparing the progressing density between T1 and T2, significant differences in BMD_a_ were observed for the three measurement methods (*p* = 0.004, 0.004, and 0.009 for Method A, B, and C, respectively). The mean density enhancement rates were 35.85% ± 51.99%, 35.61% ± 51.75%, and 37.59% ± 56.41% for Method A, B, and C, respectively. When comparing the differences in measurement values between the three methods, no statistical difference was observed for BMD_a_ (*p* = 0.536 at T1 and *p* = 0.689 at T2) or density enhancement rate (*p* = 0.681).

In unilateral cases, the BMD_a_ at T1 (BMD_aT1_) was 100.01% ± 46.98%, 102.47% ± 50.33%, and 102.78% ± 58.83%; the BMD_a_ at T2 (BMD_aT2_) was 123.17% ± 56.38%, 125.93% ± 57.82%, and 125.85% ± 61.62%; and the density enhancement rate was 36.37% ± 54.51%, 36.60% ± 53.41%, and 38.25% ± 58.38% for Method A, B, and C, respectively. No significant difference was observed for BMD_aT1_ (*p* = 0.524), BMD_aT2_ (*p* = 0.572), or density enhancement rate (*p* = 0.725) when comparing the differences among the three methods. In bilateral cases, the BMD_aT1_ was 98.24% ± 62.85%, 99.19% ± 63.97%, and 97.06% ± 68.63%; the BMD_aT2_ was 113.39% ± 43.53%, 112.34% ± 45.00%, and 108.09% ± 43.88%; and the density enhancement rate was 33.41% ± 41.41%, 30.94% ± 46.47%, and 34.46% ± 49.91% for Method A, B, and C, respectively. No significant difference was observed for BMD_aT1_ (*p* = 0.066), BMD_aT2_ (*p* = 1.000) or density enhancement rate (*p* = 0.66) when comparing the differences among the three methods.

**Figure 5:**
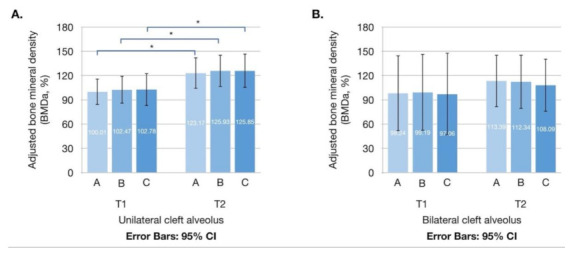
Error bars of adjusted bone mineral density (BMD_a_) of grafted tissue at T1 and T2 in individuals with (**A**) unilateral alveolar cleft and (**B**) bilateral alveolar cleft. Significant differences among the three methods were observed in unilateral alveolar cleft (*n* = 33), but no significant differences among the three methods (*p* = 0.176 for all three methods) were observed in bilateral alveolar cleft. Stars indicate significant differences in BMD_a_ between T1 and T2: *, *p* < 0.05.

When comparing the progressing density between T1 and T2, significant differences in BMD_a_ were observed for the three measurement methods in unilateral cases (*p* = 0.007, 0.007, and 0.012 for Method A, B, and C, respectively; Figure 5A); however, no significant differences were noted in bilateral cases (*p* = 0.237, 0.237, and 0.237 for Method A, B, and C, respectively; Figure 5B).

The volume of the grafted tissue in unilateral cases was 107.65 mm^3^ ± 97.67 mm^3^ at T1 and 103.52 mm^3^ ± 94.33 mm^3^ at T2; the volume of the grafted tissue in bilateral cases was 77.68 mm^3^ ± 47.57 mm^3^ at T1 and 72.93 mm^3^ ± 31.62 mm^3^ at T2. There was no significant difference when comparing the progressing volumetric change between T1 and T2 (*p* = 0.781 in unilateral cleft, and *p* = 0.893 in bilateral cleft) (Table 2). Besides, the density enhancement rate of the grafted tissue did not show a significant relationship with the volume loss (Table 3).

### 3.3. Intrarater Reliability

**Figure 6:**
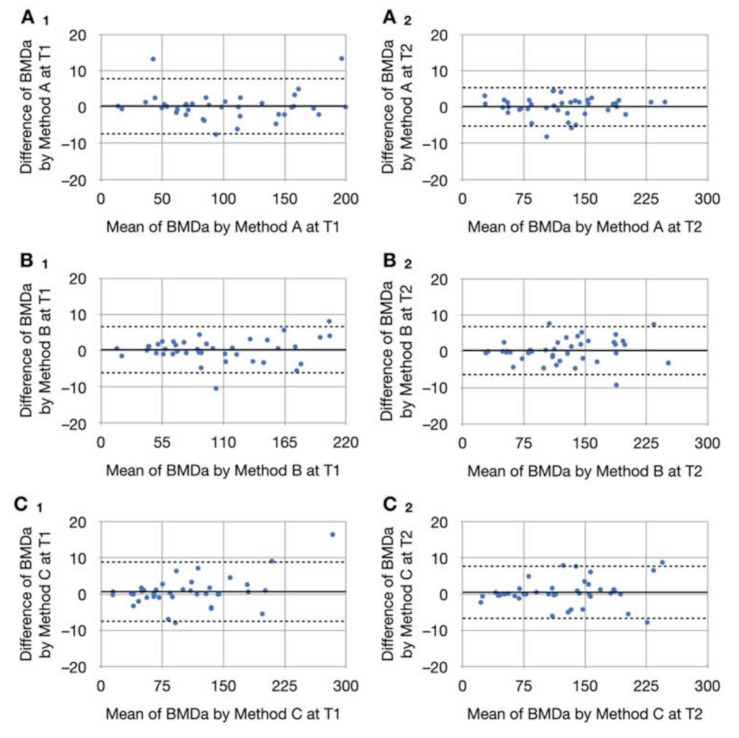
Distribution and consistency of results of two measurements using the same method and Bland–Altman plots. (**A_1_**), (**B_1_**), and (**C_1_**) plots are comparisons of two measurements conducted using Method A, B, and C, respectively, at T1; (**A_2_**), (**B_2_**), and (**C_2_**) plots are comparisons of two measurements conducted using Method A, B, and C, respectively, at T2. The mean values of differences in BMD_a_ between two measurements of grafted tissue are approximately at and evenly distributed within the zero line, indicating that the two methods produced similar results.

Our Bland–Altman analysis verified an agreement between the two examinations performed using each method at the two follow-up time points (Figure 6). Excellent intrarater reliability (ICCs > 0.9) was noted for all measurements (Supplementary Table S1) [14].

## 4. Discussion

No significant differences among the three methods were observed with respect to the BMD_a_ and density enhancement rates obtained for unilateral and bilateral alveolar cleft cases. This demonstrates that all three methods produced consistent results. However, each method still has certain advantages and disadvantages.

For Method A, the 1 mm gap relative to both adjacent teeth ensures that BMD measurements are not affected by the density of those teeth. Zhang et al. [11] proposed a similar method; the difference is that Method A involves the drawing of a maximum circle in the axial view, whereas the method proposed by Zhang et al. involves the drawing of six circles with a fixed size of 5 mm^2^ in the sagittal view. A drawback of Method A is that the surrounding cortical bone density and air density are often included in examinations of patients with large alveolar gaps. Therefore, the density of the selected area does not fully represent that of the grafted tissue.

For Method B, the selected circle contains most of the grafted area; hence, the risk of the surrounding cortical bone and air density being included is low relative to Method A. Consequently, the BMD obtained using Method B is a more objective reference. However, because the distance to the two teeth is almost tangential, but not contained, measurements must be carefully taken to avoid including sections of the teeth and therefore reducing the accuracy of the density results.

For Method C, each selected area is fixed in size and smaller than that used in Method A; therefore, the surrounding tissue is unlikely to be included, and the density of the selected area is more consistently represented relative to the other methods. However, if the alveolar gap is too wide, the selected area will not be representative of the overall density. BMD may decrease due to osteonecrosis caused by poor peripheral blood circulation [15]; furthermore, bone density may not be evenly distributed, and in patients with large alveolar gaps the results are less representative of overall BMD relative to those obtained using the other methods.

The positive and negative aspects as well as the operating precautions of the three methods are summarized in Table 4. Of the three methods, Method B theoretically produced the most representative density results for grafted tissue; however, our study revealed no statistical differences among the three methods. In addition, the correlation among all three methods was high (ICCs > 0.9), indicating that all three measurement methods can be applied in clinical settings.

Studies have investigated, but not validated, measurements of grafted tissue density in patients with orofacial clefts; between-method differences in BMD measurements were also observed [9,11,16,17,18,19]. Zhang et al. [11] obtained the following mean BMD measurements for grafted tissue: 406.51 ± 71.28 HU at 3 months postoperatively and 409.53 ± 46.37 HU at 6 months postoperatively. Benlidayi et al. [16] selected three sequential cross-sectional slices from the center of the corresponding area in CBCT images and obtained a BMD of 426.1 ± 120.1 HU with a mean follow-up period of 47.33 ± 13.79 months. Canan et al. [17] obtained BMD (273.9 ± 175.4 HU at 6 months postsurgery) measurements in five sequential axial planes with a height of 1 mm from multislice CTs. In the study of Shawky et al. [19], BMD was measured on the axial view of CTs, and the data obtained were presented as the mean of three points detected in the same axial section; these researchers obtained a mean density of 384.03 HU (214.98–549.95 HU) at 6 months postsurgery. Rychlik et al. [18] calculated BMD at every tomographic layer of the grafted tissue in CTs and obtained a grafted tissue density of 352.22 ± 84.93 HU at 6 months after ABG.

As stated, the three methods proposed in the present study have their advantages and disadvantages. Variations in density expressed as HU may be present in CBCT images [10]; therefore, the use of HU to represent BMD on unadjusted CBCT images is a drawback of the methods proposed by Zhang et al. [11] and Benlidayi et al. [16] Moreover, when children grow older, their bone density also changes [20]. To solve the aforementioned problems, we selected the density of pogonion bone marrow as the reference for calibration, thereby ensuring that our methods and collected data would be more representative of actual density than the methods proposed in previous studies. Nasoalveolar molding (NAM) solves the problem of inconsistencies in alveolar gap approximation. In our current protocol for CLP, presurgical management with NAM was used to restrict the size of the alveolar gap [12]. However, the method proposed by Shawky et al. [19] is only suitable for cases involving large alveolar gaps. Canan et al. [17] introduced a calibration method based on cerebrospinal fluid density for grafted tissue BMD analyses; this calibration method is similar to that applied in our study. However, the changes in BMD during growth must also be considered.

Although between-study comparisons of BMD results are not meaningful, changes in BMD over time can be compared across studies. Feichtinger et al. [21] discovered that the resorption of the bone graft (which is defined by volumetric change) occurred mainly within the first year after surgery, with a mean rate of 49.5%; however, a three-dimensional CT-based analysis revealed a mean bone volume that was 81% of the original value at 3 years after surgery. Several studies have assessed bone resorption using bone density. Zhang et al. [11] reported that BMD remained stable between 3 and 6 months postoperatively, and Canan et al. [17] also did not detect any significant difference in BMD among the results obtained at 3 (332.1 ± 103.0 HU), 6 (273.9 ± 175.4 HU), and 12 months (263.7 ± 183.4 HU) postoperatively.

The present study had a long follow-up period, during which the BMD_a_ of grafted tissue at 6 months and at 2 years after ABG was compared. In our study, we calculated the volume of the grafted tissue at 6 months and 2 years after the ABG procedure, and there was no significant difference between the volume at T1 and T2, which indicated that the bone volume remained stable and the results were consistent with the current consensus. Bone resorption with volume loss was published in many studies [21,22,23]; however, a significant increase in the BMD_a_ in grafted tissue was observed, and some grafted tissue BMD results were even higher than the pogonion BMD, which was compatible with the process of successful bone graft healing, i.e., osteoconduction, osteoinduction, and osteogenesis [24,25]. No correlation was observed between the density enhancement and the volume loss, thereby verifying that our ABG method was effective and that the BMD_a_ of grafted tissue increased with age.

Van der Meij et al. [23] compared unilateral and bilateral groups by using CT scans to compare the transplanted bone 1 year after ABG; their results indicated that less bone remained in the bilateral group (45%) relative to the unilateral group (70%); however, no significance testing was performed due to the small sample size. In a study by Tai et al. [22], postsurgical 1-year follow-up axial and coronal CT scans revealed no significant difference in maximal bone height, coronal volume, axial volume, maximal anteroposterior width, and maximal transverse width between unilateral and bilateral groups. In our present study, the density enhancement rate was also higher in unilateral group, but no significant difference was observed, which was consistent with the current study. Furthermore, all seven children with bilateral CLP underwent one-stage ABG, indicating that even for the more difficult bilateral cleft ABG procedure (relative to the unilateral cleft ABG procedure) [26,27,28], the progressive consolidation of grafted tissue was not inferior to that of the unilateral group.

The present study had several limitations. First, only 40 patients were examined. A larger sample size will increase the representativeness of the results pertaining to the improved BMD_a_ in the patients with alveolar clefts who underwent ABG surgery. Such a sample size will also allow us to better detect differences between the three methods. Second, only seven patients were diagnosed as having bilateral CLP, which limited our ability to compare unilateral and bilateral CLP results; the rarer occurrence of bilateral CLP may also explain the lack of such comparisons in the literature. Third, all patients received the same ABG procedure; therefore, we could not compare the difference in density enhancement rates relative to other surgical procedures. Last, we used adjusted BMD (%) in order to overcome the concern of density variations expressed as HU in CBCT images, as well as to assess the progression of BMD at different time points in cleft children; however, BMD should theoretically be the absolute value utilizing the solid phantoms, which will be improved in future studies.

We used CBCT-derived PACS as the analysis tool due to its accessibility to all surgeons; however, PACS compresses data and thus produces images of poorer quality relative to original images [29]. Furthermore, we only selected three planes as a representation of density to increase the feasibility of our proposed methods in clinical settings. In the future, 3D medical imaging software can be used to analyze images in the Digital Imaging and Communications in Medicine (i.e., DICOM) format; this allows researchers to obtain the BMD_a_ of all grafted tissue and compare the BMD_a_ results obtained using our proposed methods with the BMD_a_ of the overall graft area. Finally, bone density increases when the bone matures, and these pediatric patients with CLP will undergo further orthognathic surgery (OGS) at our craniofacial center at the age of 16–20 years [12]. Therefore, with the pre-OGS CBCTs of this group [12], we can track the progressive changes in BMD between ABG and OGS.

## 5. Conclusions

The present study reports a significant increase in BMD_a_ from 6 months to 2 years after ABG, verifying the success and feasibility of the current ABG surgical protocol. We also observed that the patients’ BMD_a_ increased by time. Furthermore, we demonstrated that our three proposed methods for measuring BMD_a_ in CBCT images can be applied in post-ABG evaluations.

## Figures and Tables

**Table 1:** Patient demographics and follow-up length.

Variables	All (*n* = 40)	Unilateral (*n* = 33)	Bilateral (*n* = 7)	*p*-Value
Age at ABG (year)	9.45 ± 1.61	9.36 ± 1.69	9.86 ± 1.12	0.467
Men (*n* (%))	24 (60.0%)	19 (57.6%)	5 (71.4%)	0.681
ABG method				
Scarpa’s fascia (*n* (%))	40 (100%)	33 (100%)	7 (100%)	—
Bone graft from iliac crest (*n* (%))	40 (100%)	33 (100%)	7 (100%)	—
Graft volume (mL) [2]	—	2.0 ± 0.6	3.8 ± 1.0	—
CBCT follow-up post-surgery				
First follow-up (month)	6.67 ± 0.82	6.60 ± 0.79	6.97 ± 0.93	0.284
Second follow-up (month)	24.04 ± 5.15	23.65 ± 4.65	25.86 ± 7.23	0.465

Data are presented as means ± standard deviations or *n* (%) and stratified by unilateral or bilateral alveolar cleft with an independent *t*-test to compare means between two groups. Categorical data are examined using the chi-squared test or Fisher’s exact test.

**Table 2:** Outcomes of cone beam computed tomography.

Variables	Method	Overall	Unilateral	Bilateral	*p*-Value
BMD_T1_ (HU)					
	A	293.88 ± 135.27	292.63 ± 135.45	299.78 ± 145.08	0.901
	B	299.50 ± 137.54	298.70 ± 137.77	303.25 ± 147.39	0.983
	C	295.84 ± 150.24	295.79 ± 150.20	296.08 ± 162.45	0.996
BMD_T2_ (HU)					
	A	360.90 ± 175.46	358.34 ± 184.54	373.01 ± 135.14	0.844
	B	366.90 ± 178.55	366.16 ± 186.93	370.41 ± 144.32	0.955
	C	361.97 ± 185.96	362.65 ± 195.78	358.78 ± 142.62	0.961
BMD_aT1_ (%)					
	A	99.70 ± 49.18	100.01 ± 46.98	98.24 ± 62.85	0.932
	B	101.90 ± 52.05	102.47 ± 50.33	99.19 ± 63.97	0.882
	C	101.78 ± 59.74	102.78 ± 58.83	97.06 ± 68.63	0.882
BMD_aT2_ (%)					
	A	121.45 ± 53.98	123.17 ± 56.38	113.39 ± 43.53	0.669
	B	123.55 ± 55.52	125.93 ± 57.82	112.34 ± 45.00	0.563
	C	122.74 ± 58.81	125.85 ± 61.62	108.09 ± 43.88	0.475
Density enhancement rate (%)					
	A	35.85 ± 51.99	36.37 ± 54.51	33.41 ± 41.41	0.893
	B	35.61 ± 51.75	36.60 ± 53.41	30.94 ± 46.47	0.796
	C	37.59 ± 56.41	38.25 ± 58.38	34.46 ± 49.91	0.874
					
Pogonion density_T1_ (HU)		312.86 ± 81.68	309.06 ± 84.55	330.77 ± 69.17	0.530
Pogonion density_T2_ (HU)		309.60 ± 88.60	303.99 ± 93.26	336.06 ± 60.40	0.391
					
Volume_T1_ (mm^3^)		–	107.65 ± 97.67	77.68 ± 47.57	–
Volume_T2_ (mm^3^)		–	103.52 ± 94.33	72.93 ± 31.62	–

Data are presented as means ± standard deviations. Differences between patients with unilateral and bilateral cleft were determined using an independent *t*-test. Difference in BMD_a_ between T1 and T2 in unilateral cases determined by a paired *t*-test: *p* = 0.007, 0.007, and 0.012 for Method A, B, and C, respectively; difference in BMD_a_ between T1 and T2 in bilateral cases determined by a Wilcoxon signed-rank test: *p* = 0.237, 0.237, and 0.237 for Method A, B, and C, respectively. BMD, bone mineral density; BMD_a_, adjusted BMD.

**Table 3:** The correlation between volumetric loss and density enhancement rate of the grafted tissue was calculated using Spearman’s correlation.

	Density Enhancement Rate (%)		
	Method A	Method B	Method C
Unilateral cleft alveolus			
Volume loss (%) *			
Correlation coefficient^®^	–0.038	0.019	0.008
*p*-value	0.844	0.921	0.966
Bilateral cleft alveolus			
Volume loss (%) *			
Correlation coefficient^®^	0.400	0.400	0.700
*p*-value	0.505	0.505	0.188

* Volume loss (%) = [(volume of the grafted tissue at T2 − volume at T1)/volume at T1] × 100.

**Table 4:** Comparison of the positive and negative aspects from each method.

	Method A	Method B	Method C
Definition	Circular zone is located 1 mm from the adjacent teeth.	The largest circular zone that exactly transects the two adjacent teeth or is tangent to the surrounding cortical bone.	A circle is drawn with a diameter of 2 mm.
Positive aspect	BMD measurements are not affected by the density of adjacent teeth.	The selected circle contains most of the grafted area. Risk of including the surrounding cortical bone and air density is low, relative to Method A.	Selected circle is fixed in size. BMD measurements are not affected by the density of adjacent teeth.
Negative aspect	The surrounding cortical bone density and air density are often included in examinations of patients with large alveolar gaps.	Easily contains adjacent tooth density or surrounding cortical bone.	Not representative of the overall density in cases with a larger alveolar gap.
Operating precautions	—	When measuring, care must be taken of to avoid including sections of the teeth.	Ensure that the distance from the circle to the two adjacent teeth is equal when measuring.

## Data Availability

The data presented in this study are available on request from the corresponding author. The data are not publicly available due to privacy.

## Supplementary Materials

The following are available online at https://www.mdpi.com/article/10.3390/jcm10215143/s1, Table S1: Inter-rater reliability for bone mineral density (BMD) measurements of alveolar defect and pogo-nion density.
