# Supplementary material for: Progressive Comparison of Density Assessment of Alveolar Bone Graft in Patients with Unilateral and Bilateral Cleft
Source: J Clin Med. 2021 Nov 1;10(21):5143. doi: 10.3390/jcm10215143 (PMC8585053; doi:10.3390/jcm10215143)
Supplement: Supplementary file 1 [file jcm-10-05143-s001.zip › jcm-1422761-supplementary.pdf]

Supplementary Materials

Supplementary Table S1. Interrater reliability for bone mineral density (BMD) measurements of alveolar defect and pogonion density.

**Parameters**	**Intraclass Correlation Coefficient**	
	**1st follow-up**	**2nd follow-up**
BMD by Method (A)	0.997 (0.994–0.998)	0.999 (0.998–0.999)
BMD by Method (B)	0.999 (0.998–0.999)	0.999 (0.998–1.000)
BMD by Method (C)	0.998 (0.997–0.999)	1.000 (0.999–1.000)
Pogonion density	0.996 (0.992–0.998)	0.826 (0.695–0.820)
